# Empowering health professions educators: enhancing curriculum delivery through customized e-tutorial training on fundamental digital tools

**DOI:** 10.3389/fmed.2024.1342654

**Published:** 2024-05-28

**Authors:** Naushaba Sadiq, Syeda Hanaa Fatima, Nadia Shabnam, Ayesha Rauf

**Affiliations:** National University of Medical Sciences (NUMS), Rawalpindi, Pakistan

**Keywords:** curriculum, teaching, assessment process, health professions, Pakistan, e-tutorial training

## Abstract

**Introduction:**

In the dynamic landscape of education, the fusion of technology and learning, commonly termed “technology-enhanced learning” (TEL), has emerged as a transformative phenomenon. This study focuses on the imperative integration of TEL in medical education, recognizing the diverse digital literacy levels of adult learners. The exploration introduces the innovative E-Portal training program, designed to empower health professions educators with essential skills for proficiently employing digital tools in instruction.

**Methodology:**

A dedicated team of medical educationists conducted a thorough investigation into E-curriculum design and delivery, employing the Moodle Learning Management System as the foundation for the E-Portal training program. The training, spanning crucial stages such as course design, content delivery, self-paced teaching, and quality assessment, facilitated participant progression at individual paces, unlocking subsequent steps upon meeting stipulated prerequisites. A pre-training questionnaire gauged participants’ comprehension of distance learning, e-learning, synchronous and asynchronous teaching, and self-directed study. Subsequent focus group discussion post-training generated rich insights into participants’ experiences, reflections, and identified challenges.

**Results:**

The results illuminate participants’ limited adeptness with e-learning terminology, successful assimilation of components and functionalities, and heightened confidence in online teaching practices. However, discerned challenges during implementation, such as technical hurdles and engagement issues, highlight the multifaceted nature of TEL integration. While the E-Portal training positively impacted preparedness, participant feedback advocates for improvements in assessment tools, technical training provisions, and exploration of alternative Learning Management Systems.

**Discussion and conclusion:**

Study emphasizes the ongoing need for diverse training methodologies across Learning Management Systems, acknowledging the evolving nature of education and technology. This study underscores the transformative role of a tailored E-Portal training program in seamlessly integrating digital tools into medical education. The comprehensive insights provided contribute to a nuanced understanding of the advantages, obstacles, and potential avenues for enhancement in curriculum delivery through technology-enhanced learning, thereby propelling the field forward.

## Introduction

The use of information and communication technologies in teaching and learning is referred to as “technology-enhanced learning” (TEL) (1); a potent phenomenon that has emerged from the convergence of technology and education in today’s fast-paced world (2). This ground-breaking approach to education is transforming conventional teaching strategies, and giving students never-before-seen chances to gain knowledge, skills, and competences in creative ways.

The demand for digital competency among educators has become increasingly imperative in response to evolving educational paradigms (3).

The imperative to embrace TEL in medical education remains strong, driven by the growing demand for innovative learning approaches, the increasing prevalence of digital natives among student populations, and the expanding scope of virtual healthcare delivery (4). By understanding and addressing the barriers to TEL integration, educators and institutions can unlock the full potential of technology to enhance teaching and learning experiences, empower learners, and shape the future of medical education (4).

As educators strive to harness the power of technology to meet the evolving needs of learners and adapt to changing educational landscapes, they encounter a myriad of challenges that must be navigated. From logistical hurdles such as limited access to technology infrastructure and resources to pedagogical considerations such as ensuring the quality and effectiveness of digital learning experiences, the journey towards integrating TEL is fraught with obstacles. Moreover, the rapid pace of technological innovation presents its own set of challenges, as educators must grapple with staying abreast of emerging trends and technologies while maintaining a focus on pedagogical principles and educational best practices (5).

The implementation of a TEL in a learning environment necessitates a novel approach that significantly impacts pedagogical and instructional design (6). Consequently, alternative teaching methods and engaging learning activities are required to effectively facilitate learning in this context (7). This adaptation entails that educators must adjust their instructional approaches while upholding consistent learning standards (8, 9). Moreover, since the efficacy of teaching is partially contingent upon educators’ proficiency in utilizing technology (7), they must actively acquire technological skills and have opportunities for experimentation and evidence-based evaluation (6, 8).

Another challenge inherent in incorporation of TEL in teaching sessions is the increased demand for coordination from educators (10). In this new instructional setting, educators must navigate between physical and virtual spaces, executing various operational tasks within the teaching and learning platform. Consequently, educators experience heightened cognitive load, characterized as hyper-zoom or hyper-focus (7, 10).

Nonetheless, the prevalence of limited digital proficiency presents a significant impediment that compromises the efficiency of teaching among faculty members. This challenge is echoed in the findings of several studies (11).

In the context of developing countries such as Pakistan, the utilization of TEL has demonstrated notable advancements; however, several impediments persist, impeding the efficacy of Technology integration within education (12). Research conducted within the Pakistani educational milieu indicates that students frequently exhibit enhanced performance on digital platforms compared to traditional methods (13). Conversely, studies suggest a deficiency in the digital competence of educators concerning the development of pedagogically sound lessons (14), notwithstanding the pivotal role of teachers in effectively integrating TEL within the classroom environment (15). Educators have delineated significant hurdles in the implementation of e-learning initiatives in Pakistan, many of which resonate within the domain of online medical education. These obstacles encompass deficiencies in instructional design, recurrent power outages, faculty resistance to embracing novel teaching methodologies, and adherence to entrenched sociocultural norms (16).

Hence, it is incumbent upon higher educational institutions to prioritize the organization of training sessions aimed at cultivating digital competence among teachers (17).

### Objectives

The primary objective of this project encompasses several key elements aimed at enhancing the utilization of Technology-Enhanced Learning (TEL) among faculty in health professions education. Firstly, the article seeks to assess the baseline knowledge and self-assessment of health professions educators regarding fundamental TEL components. This involved recognizing their existing understanding and proficiency in utilizing technology for educational purposes. Subsequently, the project aims to improve the teaching proficiency of these educators in delivering essential TEL components to undergraduate and graduate students. This enhancement was facilitated through the development and implementation of a personalized e-tutorial tailored to educate faculty members on the fundamentals of incorporating technology into their teaching methodologies.

Furthermore, this research intends to gather valuable feedback from health professions faculty regarding the usability of the e-tutorial and the challenges they encounter when integrating TEL into their instructional practices. This feedback can be instrumental in refining the e-tutorial and addressing any barriers faced by educators in adopting TEL effectively.

Aligned with these objectives, the project revolves around identifying strategies for leveraging technology to enhance instruction in resource-constrained environments. Additionally, it seeks to extract insights from the experiences of educators to inform effective faculty development initiatives, provide practical guidance applicable to similar educational contexts globally, and contribute to the formulation of improved policies and support mechanisms for technology integration in education. Through these objectives, the project aims to foster advancements in health professions education by empowering educators with the necessary knowledge and tools to effectively utilize technology in their teaching practices.

### Methodology

#### Ethical considerations

Behavioral and Ethical Research Approval (BERA) guidelines for ethics were followed (18). To maintain the confidentiality of data, all the participants were given codes for quantitative data collection, and questionnaires were tagged with the same codes. Google forms were used for collecting quantitative data. Participants were allowed to skip the demographic details. The Studies involving human participants were reviewed and approved by the institutional review board of National University of Medical Sciences (NUMS). Written informed consent to participate in this study was provided by the participants. Consent was taken from each participant both for the quantitative and qualitative data. Researcher bias was controlled as three researchers were involved in the thematic analysis of qualitative data. A new email account was used for collecting google form responses.

## Methods

It is an interventional study having both quantitative and qualitative portions. A team consisting of one biostatistician, and three medical educationists, conducted this study. Team identified key components of E-curriculum design and delivery through literature search, with a particular focus on instructional and assessment strategies. One member of the Design and Technology (DT) department of the university was involved in training researchers for creating a customized e-tutorial on LMS (open this up) tailored for faculty training. In the initial phase, the DT personnel provided training to the medical educationists who were developers of this tutorial, on all aspects of Moodle, including synchronous and asynchronous teaching features like Big Blue Button and assessment process like Multiple Choice Questions (MCQs), and Short Answer Questions (SAQs), so that they would find it easy to develop the e tutorial.

### Sampling

Forty volunteering Health Professions Educators, of various departments of the university were recruited in the study, they all gave the consent as well. All were assistant professors and above, with more than 5 years of teaching experience.

### Data collection

Data was collected in two phases. A survey concentrating on participants’ knowledge and comprehension regarding the appropriate utilization of available technologies was distributed using Google Forms before the training. The questionnaire distributed had 6 main domains with few subdomains to which participant response was required. For designing questionnaires extensive literature search was done, Questionnaire was designed based on the findings of research. First draft was sent to six experts from the field of technology and medical education. The suggestions were incorporated and sent again to experts for reconfirmation before finalization of the questionnaire. Pilot testing was done with limited number of participants.

The Second phase of data was collected through a focus group discussion (FGD) once the educators had completed the online tutorial at their convenience within a stipulated time. FDG conducted with a gap of 2 months after e-tutorial with the understanding that faculty had utilized this e-tutorial experience in their teaching learning activities as well.

The formulation of the focus group guide involved a comprehensive review of relevant literature and consultation with experts in the field in addition to some input linked to the e-tutorial. Validation of the guide was achieved through soliciting input from these experts. Subsequently, participants were invited to engage in the focus group discussion at a predetermined date and time. Out of a total of 40 potential participants, eight faculty members representing multiple departments, provided consent to take part in the discussion and were available during the data collection session. To uphold confidentiality, participants were assigned unique codes. The interaction between three researchers and the participants was audio-recorded to facilitate accurate documentation. The diverse perspectives expressed by participants contributed significantly to the depth of data collected for the project. Following data collection, transcription was conducted by the Principal Investigator. To ensure rigor and reliability, the transcribed data was independently reviewed by the Principal Investigator (PI) and two additional researchers (AR) who were not affiliated with the project as authors consolidating the triangulation process and enriching the overall validity of the findings. Subsequent thematic analysis was conducted by the same team of PI and two ARs to extract key themes from the qualitative data. This step was implemented to mitigate potential researcher bias and ensure methodological integrity.

## Data analysis

Quantitative data was analyzed through SPSS version 23. Descriptive analysis was done. Qualitative data was analyzed through thematic analysis.

### Designing of e-tutorial

The Learning Management System (Moodle) was utilized as the interface for the e-tutorial. The e-tutorial was structured such that content progressed from basic to advanced and simple to complex. However, once initiated, participants could progress through the tutorial at their own pace but within a 30 day period. Each subsequent step would unlock only when the participant had completed the required readings, watched videos, accessed other learning resources, and successfully passed a small MCQ test (See Appendix 1). Upon completion of the entire course, participants could download their E-certificates.

To facilitate understanding, the researchers created brief screen recordings demonstrating how to use various tools like Big Blue Button and design MCQs and SAQs on Moodle. Additionally, they provided YouTube links showcasing teaching and learning e-strategies such as voice over presentations, the utilization of screen recordings and use of software and apps. Relevant scholarly articles and excerpts were uploaded at relevant steps of the e-tutorial, to enhance participant understanding and reinforce concepts. Following were the sections of the e-tutorial; details of which are given in Appendix 1

- Preamble
- Wishlist of designing an online course
- Snapshot (Table of specifications)
- Dynamic and real time delivery of content
- Teaching at your pace
- Quality check for learning
- New tools

## Results

### Quantitative results

The Questionnaire had three parts. The First part focused on the participants’ understanding of different terminologies related to Technology Enhanced Learning. Each question was of the selected response type. The Second part was a self-assessment consisting of questions on competent use of different e-learning tools. The Third part inquired about participants’ viewpoints regarding formal inclusion of e-learning in the curriculum.

Collectively, the survey results indicated that participants in this study generally recognized the importance of technology-enhanced learning but displayed varying levels of understanding regarding key terminologies. While a majority associated “Distance Learning” with technology-enabled knowledge updating, there was a notable percentage considering it as another term for “E-Learning.” Additionally, participants demonstrated an understanding of “E-Learning,” as online communication between teachers and students. In terms of self-study, participants overwhelmingly favored the option of “non-timetabled self-study” outside the classroom. However, none chose “scheduled classroom study.”

Regarding teaching methods, synchronous teaching, characterized by real-time instructions, was the preferred approach, while asynchronous teaching with a time gap between instructions and responses was less favored. Participants exhibited a spectrum of knowledge and experience with online teaching, with many feeling they had average to good proficiency but still required some guidance.

These findings emphasize the need for clarity in use of the correct nomenclature in technology-enhanced learning and highlight the importance of professional development to enhance educators’ digital competence. Following tables; Tables 1–11 summarize the results of the survey questionnaire.

**Table 1:** Frequency of participants opting different responses about “distance learning.”

Option	Number of responses with percentages (*N* = 40)
Another name for E-learning	14 (35)
(Use of technology to update student’s knowledge through online learning)	18 (45)
Use of technology to address distance between student and teacher	8 (20)

**Table 2:** Frequency of participants opting different responses about e-learning.

Option	Number of responses with percentages (*N* = 40)
(Simply a broadcast of documents in electronic format to students via the Internet)	2 (5)
Another name for distance learning	6 (15)
An online communication between the teacher and the student which can be used in a classroom or an online setting	32 (80)

**Table 3:** Frequency of participants opting different responses about “self study.”

Option	Number of responses with percentages (*N* = 40)
Study of topic as directed by teacher and is time-bound	5 (12.5)
(Non-timetabled study hours spent outside the classroom for learning purposes, e.g., for assignments, group work, class test)	35 (87.5)
A scheduled time to study within a classroom for learning purposes	0

**Table 4:** Frequency of participants opting different responses about “synchronous teaching.”

Option	Number of responses with percentages (*N* = 40)
(Instructions are provided on the spot, as in face to face teaching)	38 (95)
There is defined time limit given by teacher to submit responses.	2 (5)
There is time gap between the instructions provided and response of the learners	0

**Table 5:** Frequency of participants opting different responses about “asynchronous teaching.”

Option	Number of responses with percentages (*N* = 40)
(There is time gap between the instructions provided and response of the learners)	38 (95)
Instructions are provided on the spot, as in face-to-face teaching	2 (5)
Both teacher and student must be online at the same time	0

**Table 6:** Frequency of participants opting different responses about “knowledge and experience of online teaching.”

Option	Number of responses with percentages (*N* = 40)
Poor - No knowledge & no practical experience of the subject	2 (5)
Fair - Some knowledge but no practical experience of the subject	12 (30)
Average - Knowledge and practical experience but require some guidance	17 (42.5)
Good - Knowledge and practical experience, do not require guidance	6 (15)
Excellent - Have knowledge & practical experience with the ability to start a new initiative	3 (7.5)

**Table 7:** Mean rating of responses of questions related to participants’ perceptions regarding their own competencies and expertise.

Question	Mean rating
Using technology and available software for designing an online course	3.52
Knowledge of all elements of the curriculum that should be included in an online course	3.65
Designing a synchronous teaching session (Real-time)	3.65
Designing an asynchronous teaching session (preparing a session)	3.55
Designing online assessment for students	3.25
Conducting a synchronous teaching session (Real-time)	3.35
Conducting an asynchronous teaching session (prepare and upload)	3.45
Conducting student assessment online	3.25
Providing feedback to students online	2.65
Uploading learning resources on a web portal	3.6
Google classroom	2.85
Zoom	2.75
Microsoft teams	2.975
WhatsApp	2.725
Moodle	3.15

Likert scale questions where 5 means excellent and 1 means poor.

**Table 8:** Mean rating of responses of participants about their willingness for including online teachings.

Statement	Mean (average) rating
Online teaching should be made part of regular student teaching	2.95
Online teaching should be used in addition to face-to-face teaching sessions	3.65
I will use online teaching to complement my face-to-face teaching even after COVID threat is over	2.65

**Table 9:** Frequency of responses of participants regarding academic issues.

Academic issues	Number of participants
Lack of effective assessment strategies	8
Lack of students’ active participation during teaching sessions	12
Poor adaptability of students to online teaching	6
Poor adaptability of teachers to online teaching	2
Lack of courses/content availability	1
Lack of faculty enthusiasm during teaching sessions	1
Any other (unspecified academic issues)	3
None (no specific academic issues mentioned)	7

**Table 10:** Frequency of responses of participants regarding technical issues.

Technical issues	Number of participants
Poor connectivity	21
Non-availability of training program prior to the launch of online teaching	10
Any other (unspecified technical issues)	4
Non-availability of campus management system (LMS/CMS)	3
None (no specific technical issues mentioned)	2

**Table 11:** Frequency of responses of participants regarding administrative issues.

Administrative issues	Number of participants
Scheduling issues of online teaching sessions	7
Absence of online teaching policy	8
Mal distribution of faculty workload	5
Lack of Interdepartmental coordination	6
Any Other (unspecified administrative issues)	5
Absence of designed faculty time	3
Absence of accountability and record-keeping	2
Faculty resistance	1
None (no specific administrative issues mentioned)	3

Tables 1–6 are giving the frequency as well as percentages of responses in each dimension.

Table 7 shows the mean rating and mode of responses while Table 8 summarizes the view points of the participants regarding inclusion of technology enhanced teaching in the curriculum. Overall results shows that participants seem reluctant in making online teaching as a regular part of students’ teaching, they preferred using it as an additional mode.

Table 9 summarizes the categories of academic issues mentioned by participants and the number of participants who mentioned each issue. Most common issue highlighted by the participants was “Lack of students’ active participation during teaching sessions.”

Table 10 displays the categories of technical issues mentioned by participants and the number of participants who mentioned each issue. Poor connectivity is the most frequently mentioned issue.

Table 11 summarizes the categories of administrative issues mentioned by participants and the number of participants who mentioned each issue. The absence of an online teaching policy is the most frequently mentioned administrative issue.

### Qualitative results

The themes that emerged in focus group discussion can be summarized in the Table 12.

**Table 12:** Qualitative results showing themes, codes and examples of relevant quotes.

S.no	Theme	Codes	Quotes
1.	Understanding of Terminologies related to TEL	Familiarity	“If you ask me, I never gave any significance to these terms before this training on the e-portal; now I can definitely say that I know for sure what these terms mean.”
		significance	
		Readiness for future	
		Relevance	
2.	Benefits of e-tutorial training	Knowledge of components	“I think my E teaching has systemized after getting trained through this e portal, like now I know the components of a curriculum that is delivered through e-learning, the requirements and the assessment strategies.”
		E learning platforms	“Before that training on the portal, we did not have any knowledge about any e-learning platform or even about Moodle. Now we are using Moodle to manage our e-learning, and the portal helped us a lot in knowing different functions like assessments and quizzes and presentations upload.”
		Functions of Moodle	
		Synchronous learning tool	
		Access, ease and availability	
		Organized learning	
		Back up data	
		Made work easy	
		Multiple tools and functions	
3.	Improved confidence	Confidence	“I will specifically comment on the assessment part; while using Moodle, we had to send our material to the IT department and request them to upload it, but now we are confident and comfortable in doing both”
		Enjoyment	
		Comfort	
		More interesting	
		Suitable and effective	
4	Problems faced during implementation of online teaching	Engagement issue	“The chances of cheating have increased in online teaching.” “Preparing learning materials that can engage students for online teaching definitely needs more time.”
		Increased cheating	
		Lack of policies	
		Lack of SOP for the students	
		Lack of feedback	
		Lack of training	
		Lack of expressions /body language	
5.	e-tutorial value in addressing issues	Training done	“I think the training of the portal equipped us to cater for *our lack of* knowledge and build our preparedness, but it cannot help technical issues like what to do when there are weak internet signals. Yes, we can use a synchronous tool for that matter, but we still need to look after such issues.”
		Preparedness	
6.	Propositions for Improvement	Technical training	“Assessment tools should be improved, and training should be there.”
		Detailed training	
		Improved tools	
7.	Preparedness for future situations	Hybrid lessons	“It should not be like that we are on campus and teaching in person, we forget all the training. These trainings should go parallel, and lessons should also be hybrid.”
		Training on other LMS	

**Figure 1:**
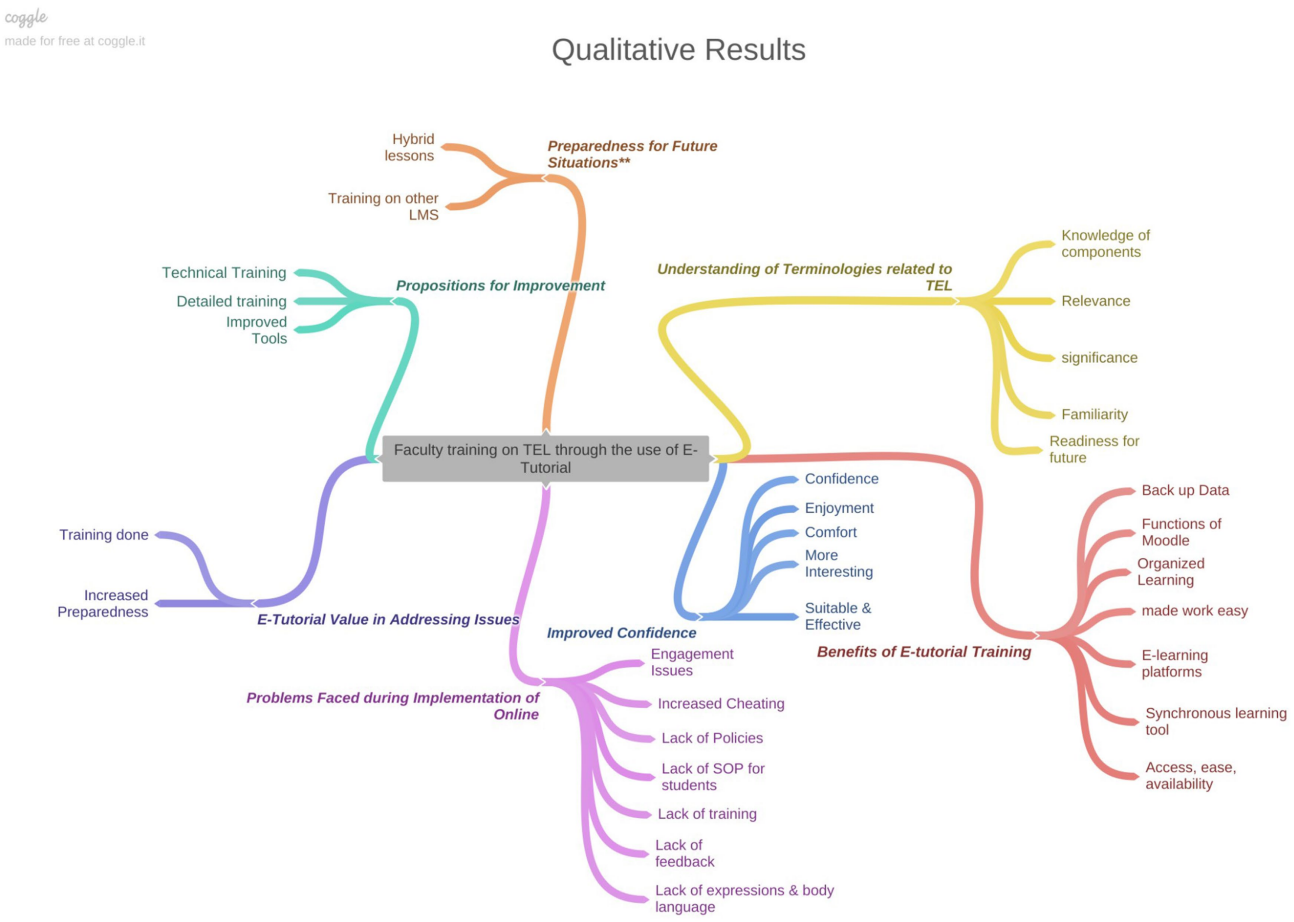
Qualitative results.

The qualitative results can be explained with the help of Figure 1.

### Understanding of terminologies related to TEL

In the context of the focus group study, participants were queried about their comprehension of terminologies pertinent to technology-enhanced learning, including synchronous learning. Post-engagement with an e-tutorial, participants reported an enhanced understanding of key terms such as synchronous and asynchronous learning, distance learning, and e-learning.

The participants expressed a heightened awareness of the significance of acquiring knowledge in these terminologies for a more profound grasp of online teaching strategies and pertinent literature related to e-learning. Notably, Participant 8 articulated this shift in perception, stating, “If you ask me, I never gave any significance to these terms before this training on the e-portal; now I can definitely say that I know for sure what these terms mean.”

These findings underscore the efficacy of targeted e-tutorial interventions in elucidating complex terminologies associated with technology-enhanced learning, thereby contributing to participants’ broader comprehension of the subject matter. The acknowledgement of the newfound importance of these terms signifies a positive outcome in terms of knowledge acquisition and contextual understanding.

### Benefits of e-tutorial training

In response to inquiries regarding their acquired knowledge from the e-tutorial, participants articulated a comprehensive understanding of various components intrinsic to e-learning and e-curriculum. This newfound knowledge encompassed insights into online learning platforms, exemplified by Moodle, and its multifaceted functions, including assessments, quizzes, and the creation of voice-over presentations.

Participants underscored the transformative impact of the training, asserting that it equipped them with innovative teaching tools such as screen recordings and voice-over presentations. The structured design of the e-portal, coupled with the accessibility of resources, emerged as pivotal factors facilitating their learning experiences.

Participant 3 elucidated, “I think my E teaching has systemized after getting trained through this e portal, like now I know the components of a curriculum that is delivered through e-learning, the requirements and the assessment strategies.” This statement underscores the tangible impact of the e-tutorial on the organization and structuring of the participant’s electronic teaching methods.

Participant 5 corroborated these sentiments, stating, “Before that training on the portal, we did not have any knowledge about any e-learning platform or even about Moodle. Now we are using Moodle to manage our e-learning, and the portal helped us a lot in knowing different functions like assessments and quizzes and presentations upload.” This acknowledgment highlights the pivotal role of the e-tutorial in introducing participants to previously unfamiliar e-learning platforms and subsequently enhancing their utilization of Moodle for effective e-learning management.

### Improved confidence

Following their participation in the e-tutorial, participants conveyed an enhanced sense of confidence in orchestrating online teaching sessions and adeptly employing diverse tools for online learning. This newfound assurance extended to their proficiency in conducting both synchronous and asynchronous teaching sessions, as well as in the nuanced tasks of designing and uploading e-assessments, coupled with the provision of constructive feedback.

Participant 6 elucidated on the impact of the training, stating, “I will specifically comment on the assessment part; while using Moodle, we had to send our material to the IT department and request them to upload it, but now we are confident and comfortable in doing both.” This remark signifies a tangible empowerment regarding the independent management of e-assessment processes, a capability acquired through the e-tutorial.

Similarly, Participant 4 expressed a preference for synchronous learning and acknowledged the added allure brought about by e-learning tools, remarking, “I think I like synchronous learning more, but yeah, the e-learning tools made it more interesting, and the e-tutorial helped us a lot.” This sentiment underscores the positive impact of the e-tutorial not only in fortifying confidence but also in rendering the learning process more engaging through the incorporation of innovative tools.

### Problems faced during implementation of online teaching

During inquiries into the challenges encountered during the implementation of e-learning, participants identified several significant issues, notably technical difficulties such as weak internet signals, concerns about cheating during assessments, and the perceived necessity for more time to prepare learning resources for online teaching.

Participant 8 underscored the escalating risk of academic dishonesty in online teaching, stating, “The chances of cheating have increased in online teaching.” Participant 4 emphasized the time-intensive nature of crafting engaging learning materials, noting, “Preparing learning materials that can engage students for online teaching definitely needs more time.” Participant 2 further articulated the time constraints faced in a fast-paced working environment, particularly in the creation of voice-over presentations for online sessions.

The lack of established policies and standardized operating procedures (SOPs) emerged as another major hurdle in effective e-learning implementation, as highlighted by Student 5 and endorsed by Student 6. They noted the importance of faculty preparedness, which was addressed to some extent through the use of the portal, but stressed the need for policies and SOPs, especially for students.

Participants 2 and 3 expressed concerns about student attitudes, noting a casual demeanor, reluctance to open cameras, and a perceived lack of seriousness during online classes. This aspect, coupled with the absence of policies, hindered the effectiveness of e-learning.

Additionally, participants voiced a need for further training on providing feedback and engaging students effectively. Participant 3 proposed incorporating components in the e-tutorial focusing on learner engagement and effective feedback. Participant 7 identified student engagement as a significant challenge. Participants collectively emphasized that body language, a crucial element in live teaching, was not adequately addressed in online settings.

In conclusion, the identified challenges encompass technical issues, time constraints, the absence of policies and SOPs, and difficulties related to student engagement. The participants’ insights underscore the multifaceted nature of challenges inherent in the effective implementation of e-learning, necessitating comprehensive solutions and additional training components to address these concerns.

### e-tutorial’s value in addressing issues

When questioned about the efficacy of the e-tutorial in addressing previously mentioned challenges, participants acknowledged the role of the e-portal in enhancing their preparedness and knowledge. However, they noted that certain issues, such as technical challenges, cheating prevention, measurement of student understanding, and student engagement, were inadequately addressed by the e-tutorial.

Participant 1 recognized the training’s effectiveness in addressing knowledge gaps and building preparedness but highlighted its limitations in dealing with technical challenges. Specifically, the participant noted, “I think the training of the portal equipped us to cater for lack of our knowledge and build our preparedness, but it cannot help technical issues like what to do when there are weak internet signals. Yes, we can use a synchronous tool for that matter, but we still need to look after such issues.”

Concerns about cheating prevention were expressed by Participant 1, who stated, “I am still confused on how to cater for cheating among students.” This reflects a perceived gap in the training’s coverage of strategies to address academic dishonesty in an online learning environment.

Participant 2 pointed out the disadvantage of lacking visual cues such as body language in e-learning, making it challenging to gauge student understanding. The participant remarked, “Disadvantage is that the body language of students, expressions were not there in e-learning. I could not guess whether they are understanding or not, so could not measure the outcome. This issue is not catered in training through the e-portal.”

In summary, while participants acknowledged the benefits of the e-tutorial in enhancing their preparedness and knowledge, they identified specific challenges that were not adequately addressed, particularly in the realms of technical issues, cheating prevention, and measuring student understanding in an online context. These insights highlight the need for a more comprehensive approach to training that encompasses a broader range of challenges associated with e-learning.

### Propositions for improvement

When queried about potential improvements in the training, participants proposed enhancements in assessment tools, particularly advocating for the incorporation of subjective questions (Short Answer Questions - SAQs) in conjunction with multiple-choice questions (MCQs). Participant 2 emphasized this point, stating, “Assessment tools should be improved, and training should be there.”

Additionally, participants recommended refining technical training and expanding options within MCQs to enhance the assessment process. This indicates a collective recognition of the pivotal role of assessment tools in gauging student understanding and proficiency in an online learning environment.

Another notable suggestion from the participants was to explore alternative Learning Management Systems (LMS) beyond the current platform, with a concurrent recommendation for training on these alternative platforms. Student 3 articulated this viewpoint, stating, “I think yes, but other LMS should be explored, and we should be trained on these LMS.” This recommendation underscores a proactive approach to diversifying the technological infrastructure for online learning, potentially catering to varying instructional needs and preferences.

In summary, participants’ suggestions for improvements in the training program revolve around the refinement of assessment tools, including the integration of subjective questions and exploration of alternative LMS, accompanied by corresponding training initiatives. These recommendations align with the participants’ desire for a more comprehensive and versatile training experience in the realm of technology-enhanced learning.

### Preparedness for future situations

When questioned about their preparedness for unforeseen situations in the future, participants expressed a sense of readiness but underscored the imperative of continuous training, particularly on hybrid teaching approaches.

Participant 3 articulated this perspective, stating, “It should not be like that we are on campus and teaching in person, we forget all the trainings. These trainings should go parallel, and lessons should also be hybrid.” This assertion emphasizes the need for a sustained and concurrent approach to professional development, ensuring that training initiatives run in tandem with on-campus teaching activities. The call for hybrid lessons indicates an awareness of the evolving nature of educational practices and the necessity for educators to seamlessly integrate face-to-face and online teaching strategies.

In academic terms, the participants are advocating for an ongoing and integrated approach to professional development that aligns with the dynamic landscape of educational methodologies, with a specific emphasis on hybrid teaching approaches. This perspective reflects a commitment to adaptability and continuous improvement in response to evolving educational needs.

## Discussion

The discussion is structured around key domains emerging from both quantitative and qualitative results, with relevant references incorporated to provide a comprehensive analysis.

### Knowledge and usability of TEL

The quantitative results of the study unveil a spectrum of understanding among participants regarding key terminology associated with technology-enhanced learning (TEL). Notably, 18 participants (45%) identified “Distance Learning” as “the use of technology to update students’ knowledge through online learning.” This points to a limited recognition of the role of technology in knowledge dissemination. It is noteworthy that this understanding might be influenced by the limited usage of online platforms and digital learning tools in traditional face-to-face settings.

A parallel study conducted by Gyampoh et al. (19) where faculty revealed a similar trend. More than 60% of participants in that study expressed feelings of incompetence and unfamiliarity regarding the requirements of using TEL in their teaching practices. These findings echo the sentiments of Petrusevich (20), emphasizing the importance of clarifying terminology in the context of online education for the effective design and delivery of online courses.

The identified lack of knowledge and limited usability can be effectively addressed through training and development initiatives for faculty. Ng and Lam’s (21) study support this notion, highlighting that easy acceptance and behavioral adjustments were achieved through a well-thought-out plan. This plan, aligned with the institution’s goals, gradually introduced e-elements in various training sessions. This approach not only addresses the challenges identified in the current study but also aligns with the insights from Govender and Govender (22), cited in Liu et al. (23), indicating that even teachers with access to technology and computer competency skills may struggle to integrate technology into their teaching practices. Proficient users of 21st-century technology, as emphasized by Manzano (24), are essential for the efficient integration of technology in education.

### Problems faced during implementation of online teaching

The qualitative findings derived from the focus group discussion underscore the positive impact of the e-portal training on participants’ knowledge acquisition in various facets of e-learning. Participants reported gaining practical insights into the utilization of online learning platforms, such as Moodle, during the tutorial. The systematic design of the e-tutorial, complemented by the availability of resources and innovative teaching tools, significantly contributed to enhancing participants’ confidence. This training not only increased their proficiency with digital tools but also empowered them to conduct both synchronous and asynchronous teaching sessions effectively. This aligns with the broader perspective that teacher training programs play a pivotal role in elevating digital competence within the higher education landscape (25).

Conversely, the study identified challenges during the implementation of online teaching, with technical issues, particularly poor connectivity, emerging as a prevalent problem. This observation resonates with existing research on the challenges of online education, particularly in developing countries where inadequate internet infrastructure poses a significant hindrance (26). Additionally, challenges related to student engagement and the measurement of student understanding underscore the importance of pedagogical strategies and tools that foster active participation and effective assessment in online environments (27).

The study findings also echo the broader challenges faced by educators in the online teaching landscape, including the need to engage students effectively, manage virtual classroom dynamics, adapt course content for online delivery, overcome technical issues, and ensure sufficient student participation (28). These challenges are compounded by variations in institutional support, policies, and educator attitudes, emphasizing the complexity of enhancing the quality of online education (19, 29–31).

Furthermore, participants’ suggestions for improvement center on enhancing assessment tools and providing more robust technical training. The recommendation to incorporate subjective questions (SAQs) along with multiple-choice questions (MCQs) underscores the quest for more comprehensive assessments. Additionally, the proposal to explore a variety of learning management systems (LMS) and provide corresponding training aligns with the need for educators to adapt to diverse digital teaching environments (32). These recommendations emphasize the crucial role of continuous professional development for educators to remain abreast of evolving digital tools and teaching strategies (33).

### Preparedness for future situations

The study illuminated participants’ perceptions of preparedness for future uncertainties, particularly those precipitated by events like the COVID-19 pandemic. Participants acknowledged the significance of continuous training, specifically on various Learning Management Systems (LMS) and hybrid teaching approaches. This underscores the imperative for universities and educational institutions to invest in sustained faculty development initiatives, ensuring that educators remain adaptable in the face of evolving educational landscapes (34).

In the realm of medical education, the study suggests that administrators and educators must actively seek out innovative technologies to maintain the standards of medical education. It is imperative for medical educators to embrace emerging technologies that have the potential to shape the future of medical education (35–38). This adaptation to evolving technologies is crucial for enhancing the quality and effectiveness of medical education, especially in the context of unforeseen challenges and uncertainties.

## Conclusion

To sum up, this research has yielded significant findings on the comprehension of technology-enhanced learning jargon by the participants, the influence of e-portal instruction on their digital proficiency, and the obstacles encountered when executing remote instruction. The results highlight how crucial it is for educators in the digital age to have clear language, efficient training, and continuous professional development. They also emphasize the necessity of resolving administrative, pedagogical, and technical issues in order to guarantee the success of online learning projects.

In general, this study adds to the ongoing discussion about how education is becoming more digital and how teachers are adopting more technology- enhanced teaching strategies. It emphasizes how crucial it is to give teachers the abilities and information required to succeed in digital learning environments.

## Limitations

The sample size for focus group discussion was relatively small, and the participants were health professionals of only one institute; though from different disciplines, which may limit the generalizability of the findings.

Additionally, the study did not investigate the long-term impact of the training on participants’ teaching practices.

## Data availability statement

The raw data supporting the conclusions of this article will be made available by the authors, without undue reservation.

## Ethics statement

BERA guidelines for ethics were followed [Freedman et al. (18)]. To maintain the confidentiality of data, all the participants were given codes for quantitative data collection, and questionnaires were tagged with same codes. Google forms were used for collecting quantitative data. Participants were allowed to skip the demographic details. The Studies involving human participants were reviewed and approved by the institutional review board of National University of Medical Sciences (NUMS). Written informed consent to participate in this study was provided by the participants. Consent was taken from each participant both for the quantitative and qualitative data. Researcher bias was controlled as three researchers were involved in the thematic analysis of qualitative data. A new email account was used for collecting google form responses.

## Author contributions

NSa: Conceptualization, Writing – review & editing. SF: Conceptualization, Formal analysis, Investigation, Methodology, Writing – original draft, Data curation. NSh: Conceptualization, Data curation, Formal analysis, Methodology, Project administration, Supervision, Visualization, Writing – original draft, Investigation. AR: Conceptualization, Project administration, Writing – review & editing.
