# Supplementary material for: Empowering health professions educators: enhancing curriculum delivery through customized e-tutorial training on fundamental digital tools
Source: Front Med (Lausanne). 2024 May 28;11:1342654. doi: 10.3389/fmed.2024.1342654 (PMC11168105; doi:10.3389/fmed.2024.1342654)
Supplement: Supplementary file 1 [file Data_Sheet_1.docx]

**Appendix 1**

**Details of E-Tutorial:**

The training course on E-Tutorial was divided into the following stages

**Figure 1: Shows the component of E-Tutorial with description and Screenshot**

| **Preamble:** | | | This encompassed both the introductory message and the justification for the training |
| --- | --- | --- | --- |
| 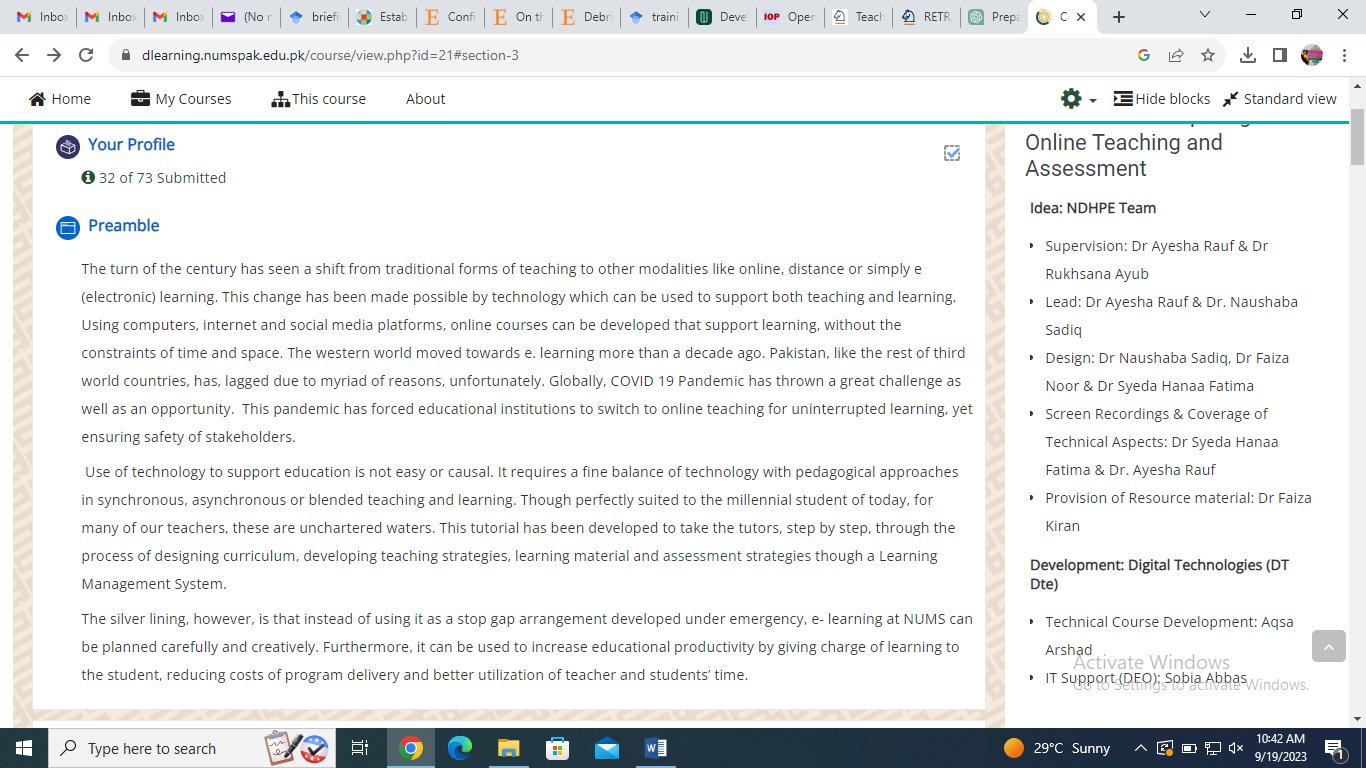 | | | |
| **Outcomes:** | | | This part highlighted the outcomes of training |
| 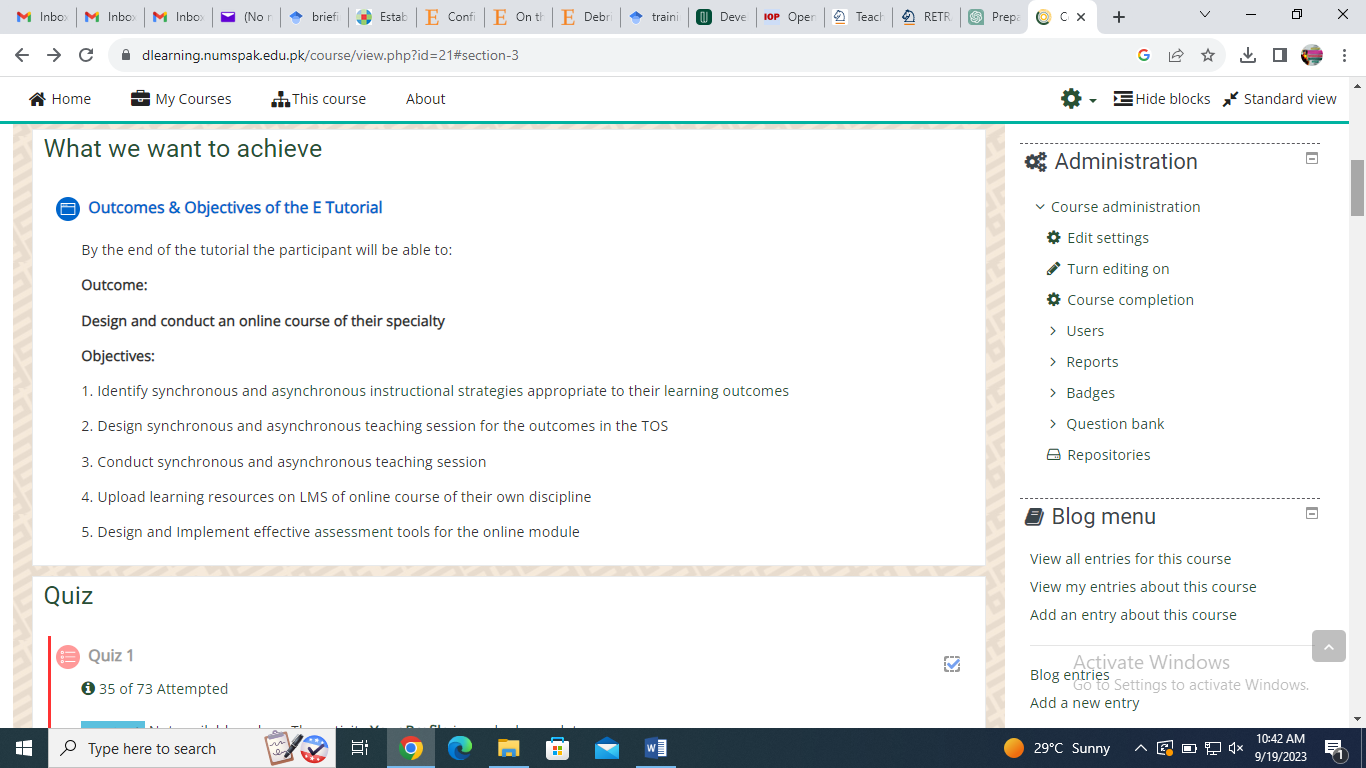 | | | |
| **Wish list for Course Design** | | Participants explored the essentials and considerations involved in designing a course effectively. | |
| 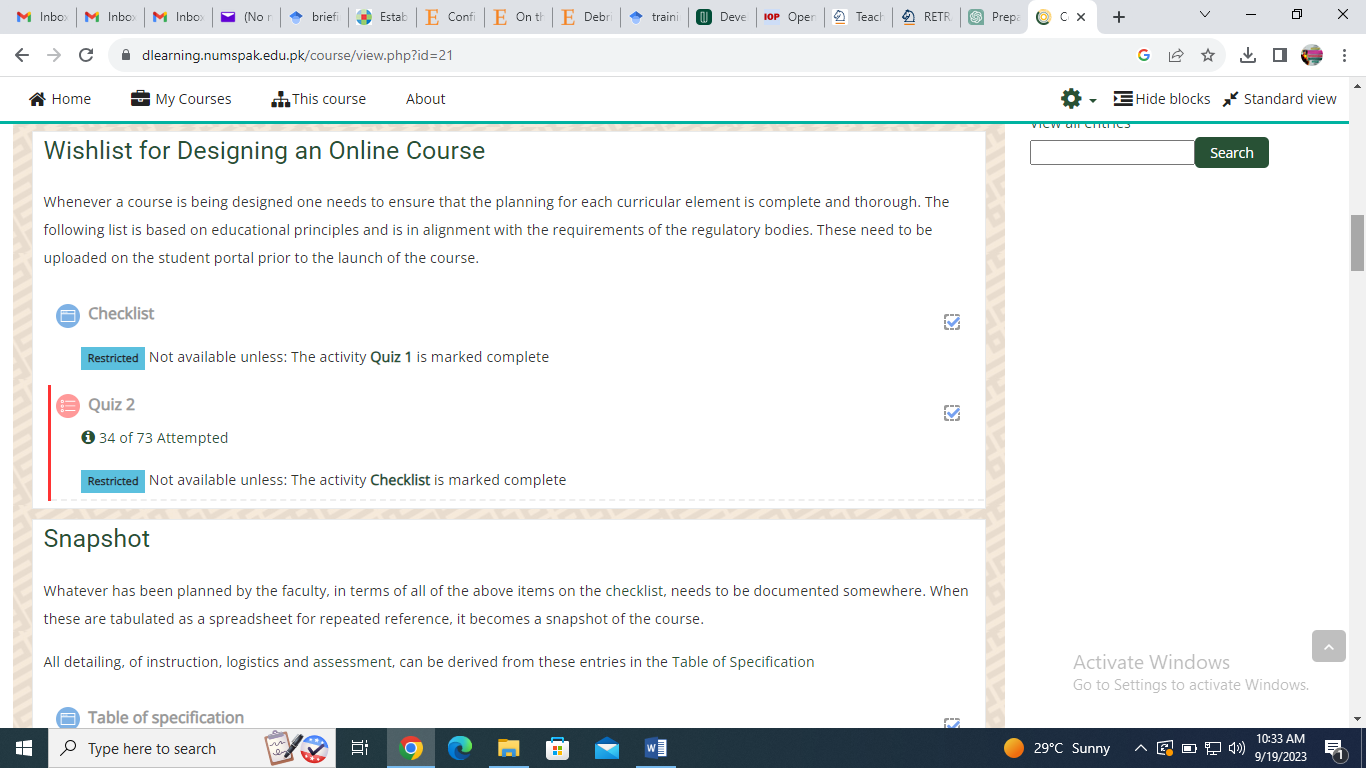 | | | |
| 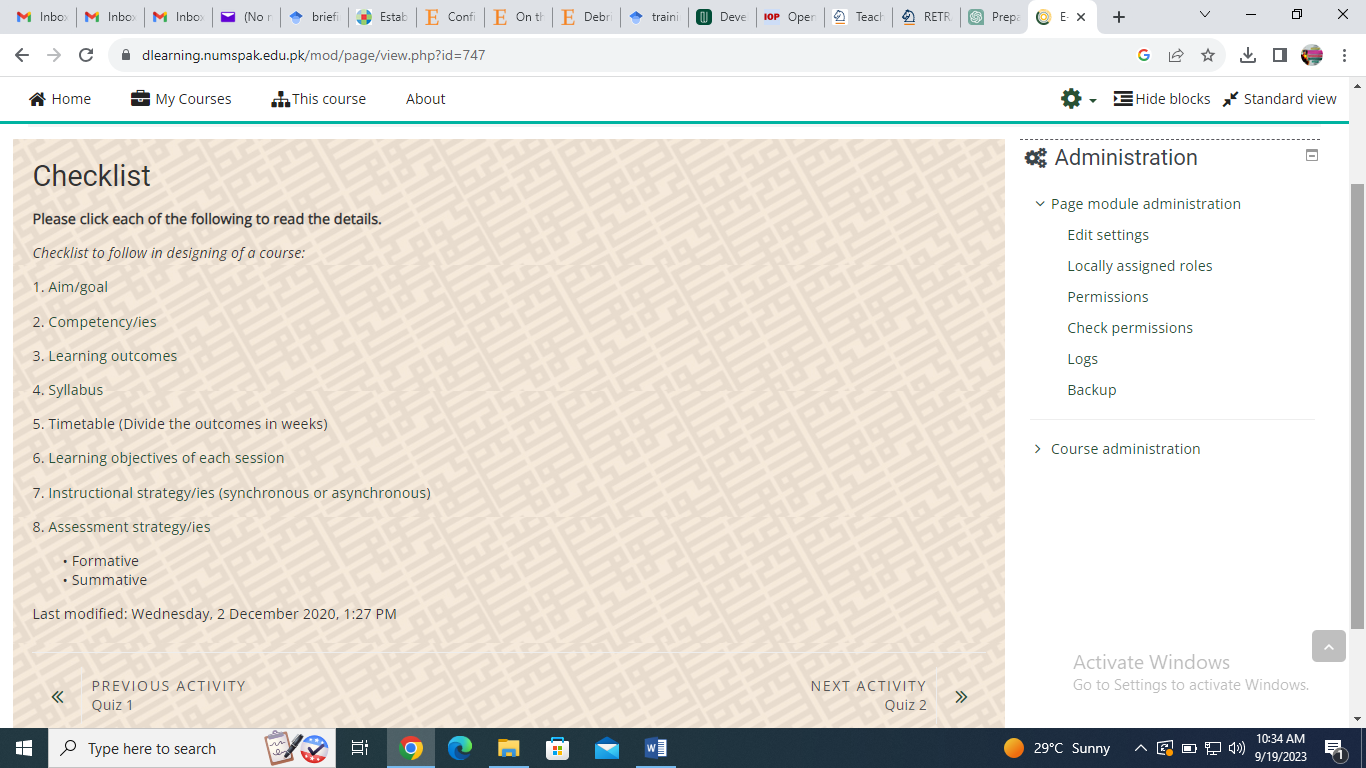 | | | |
| Each heading of checklist; when clicked opened the details and the learning resource of that particular item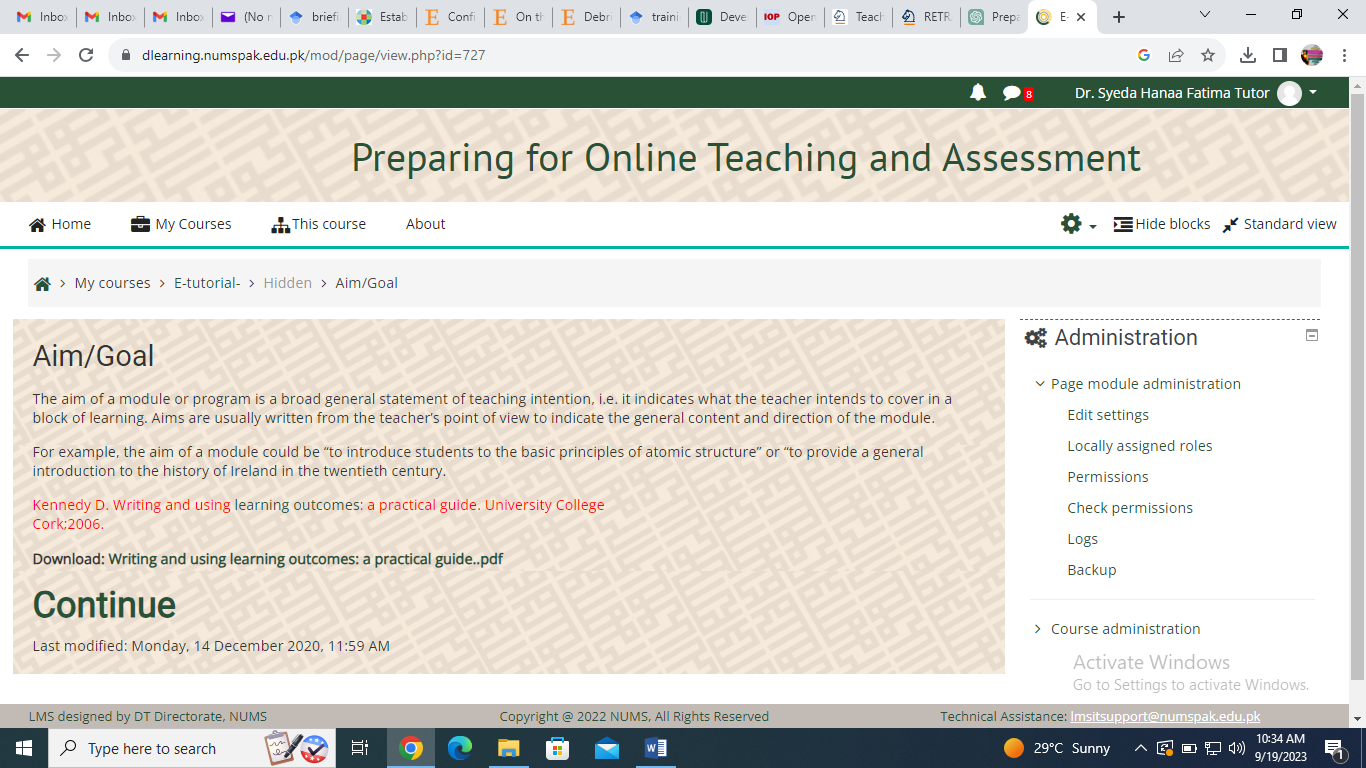 | | | |
| **Snap Shot/Table of Specifications** | This stage focused on providing participants with a comprehensive understanding of creating a detailed course outline and specifications. | | |
| 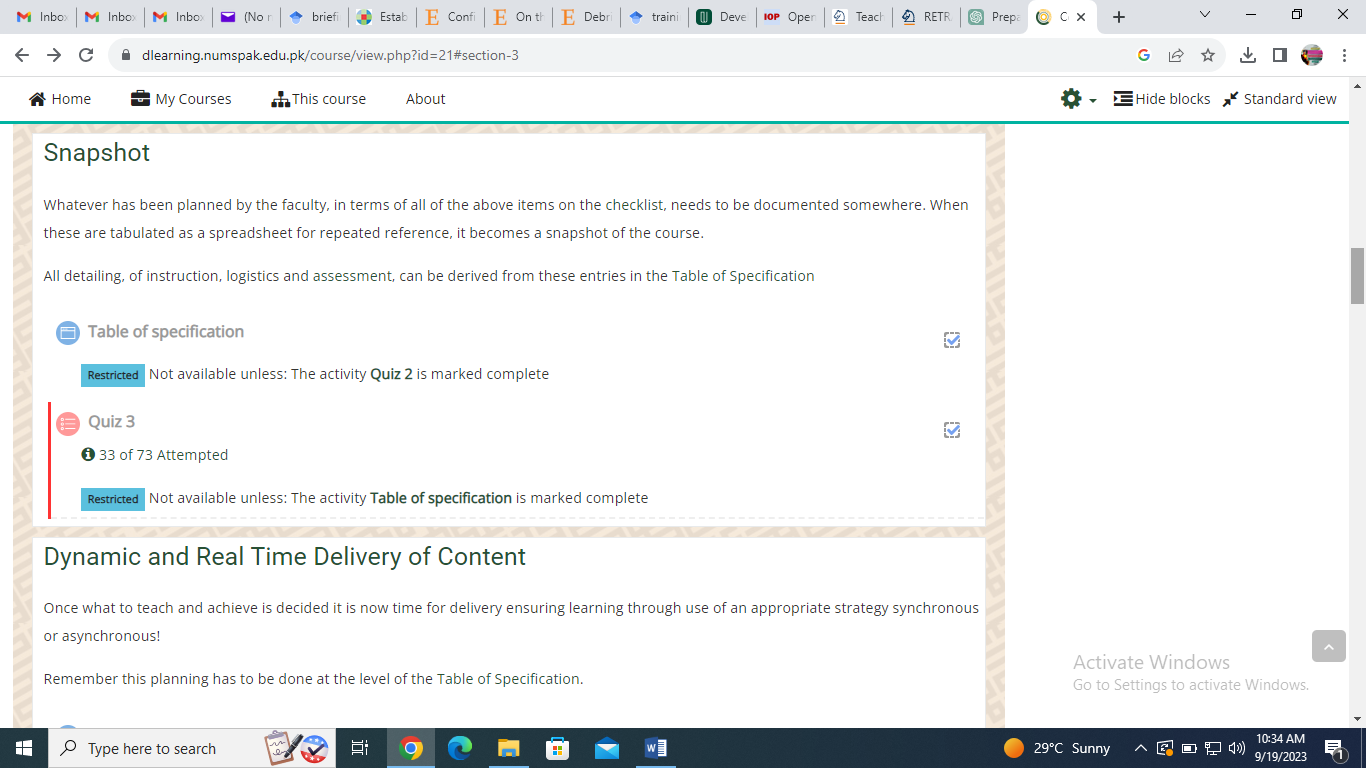 | | | |
| Details of table of specification and example opened up when heading was clicked 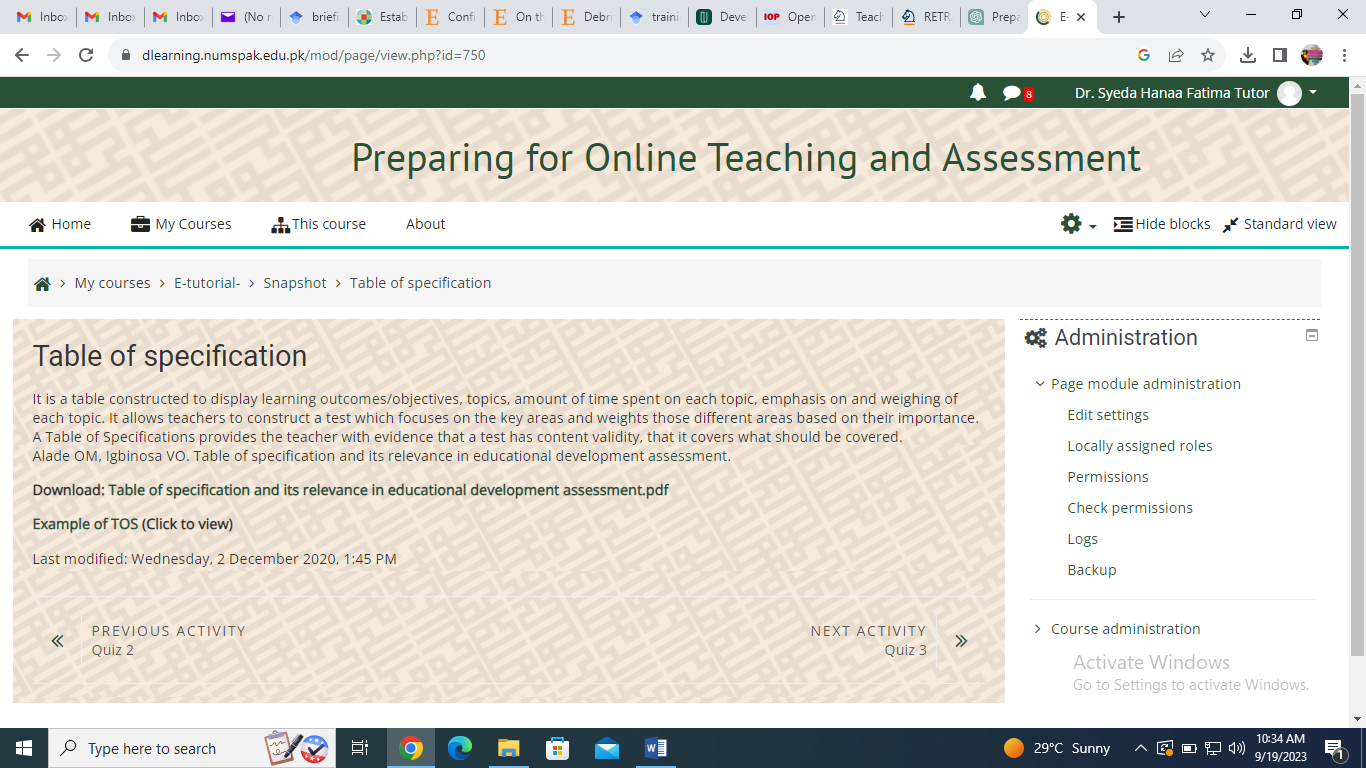  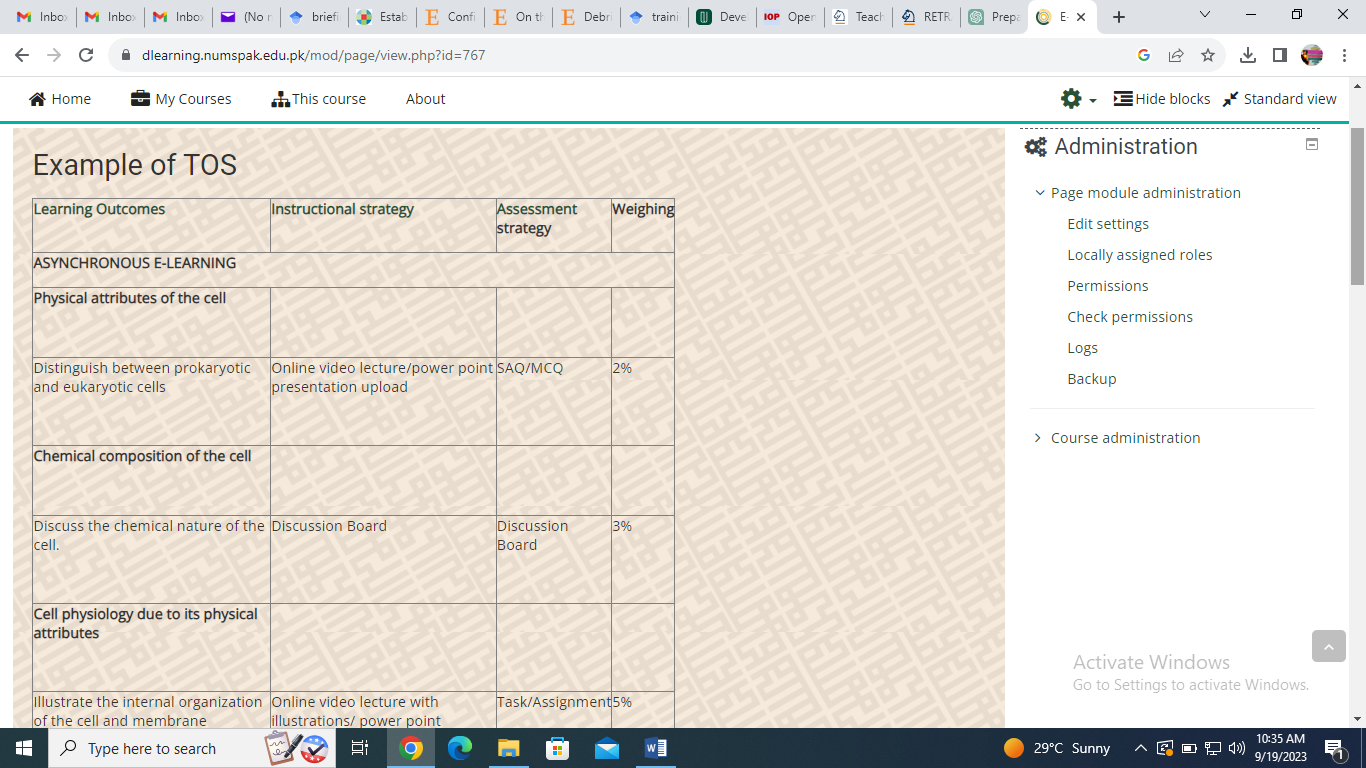 | | | |
| **Dynamic and Real-Time Content Delivery**: | Participants learned how to deliver course content dynamically and in real-time to enhance the learning experience. | | |
| 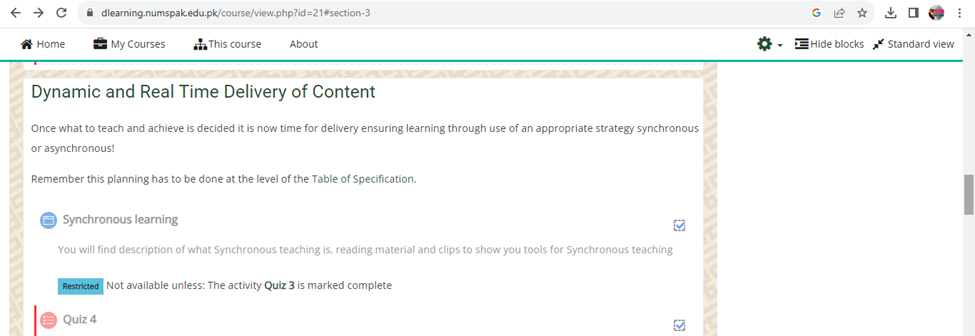 | | | |
| Details of Dynamic teachings with examples were imbedded in this section as shown in the following screen shots | | | |
| 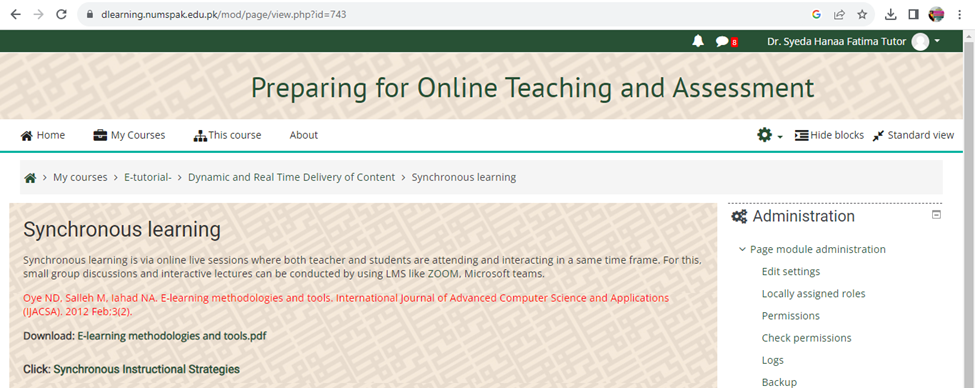 | | | |
| 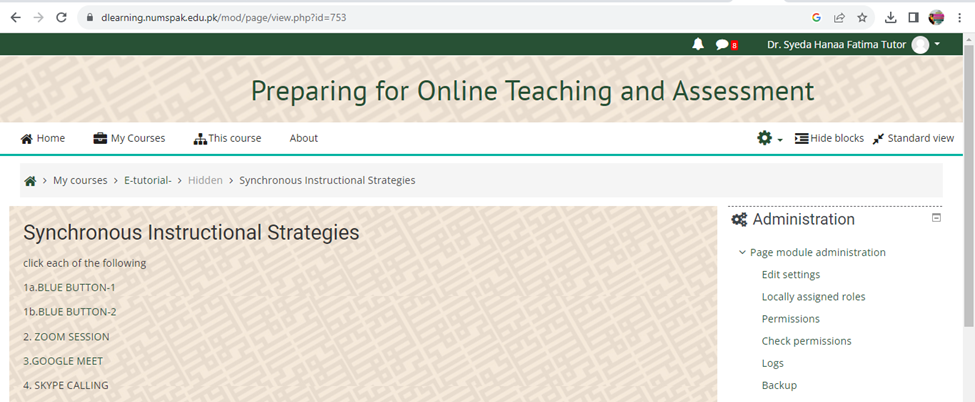 | | | |
| 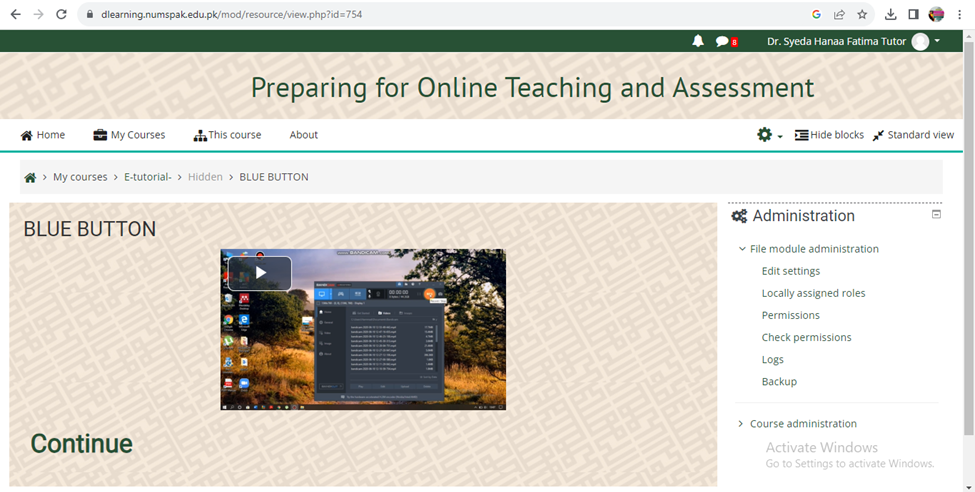 | | | |
| 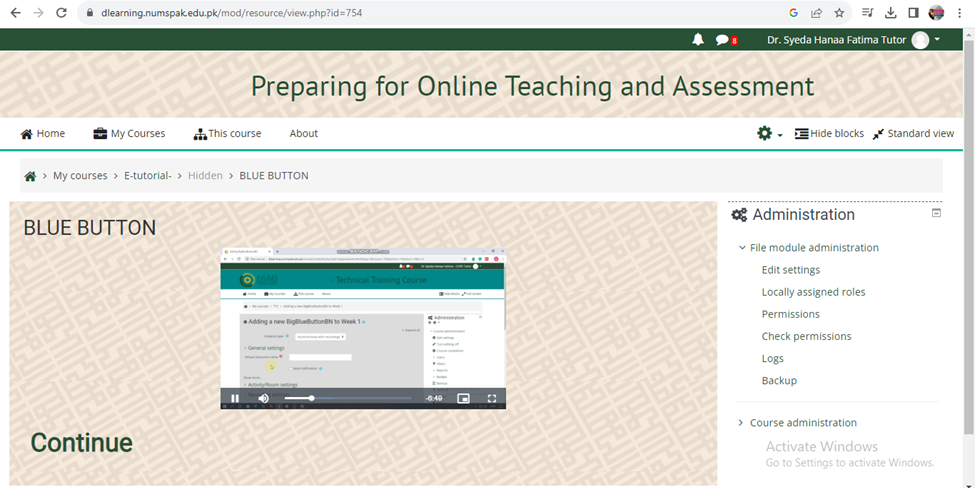 | | | |
| **Self-Paced Teaching** | This stage emphasized the importance of self-paced teaching, allowing participants to adapt their learning journey to suit their individual needs. | | |
| 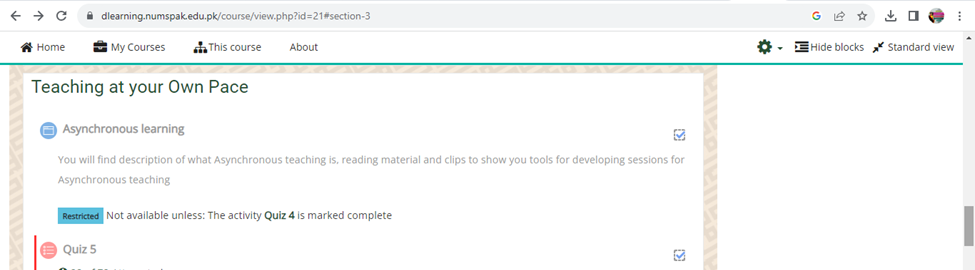 | | | |
| 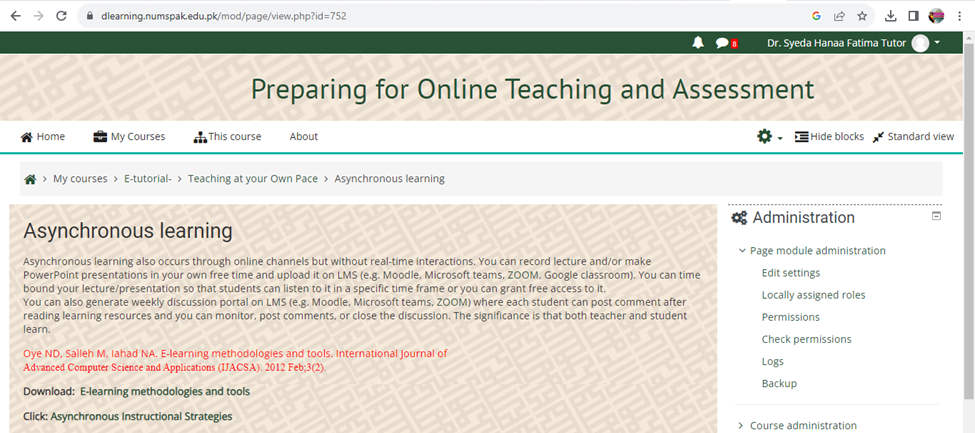 | | | |
| 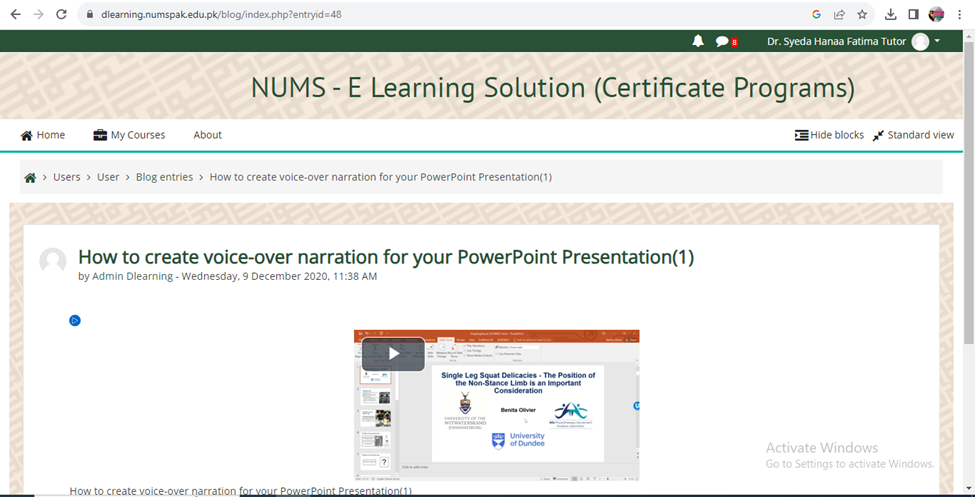 | | | |
| 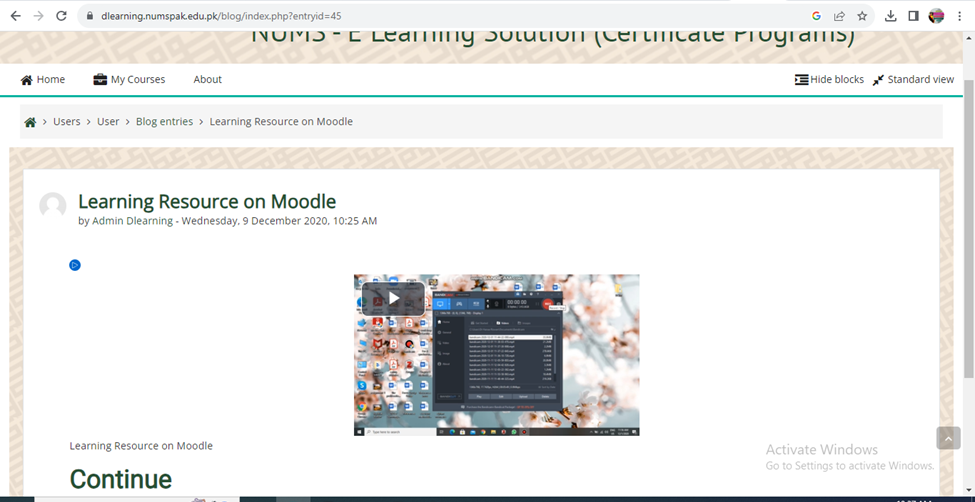 | | | |
| **Quality Check for Learning** | Participants delved into techniques for assessing and ensuring the quality of the learning experience throughout the course. | | |
| 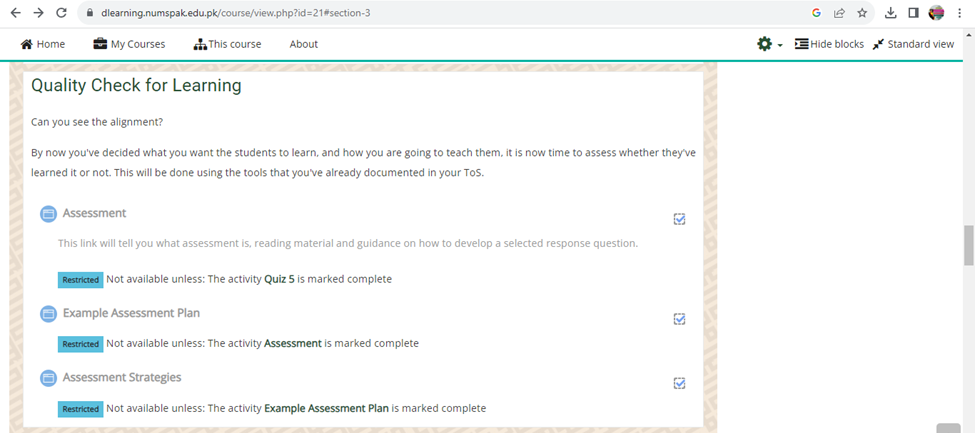 | | | |
| 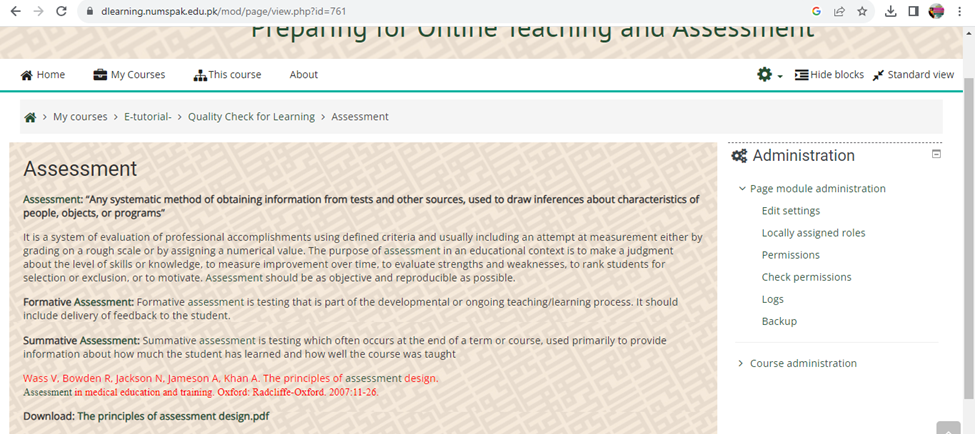 | | | |
| 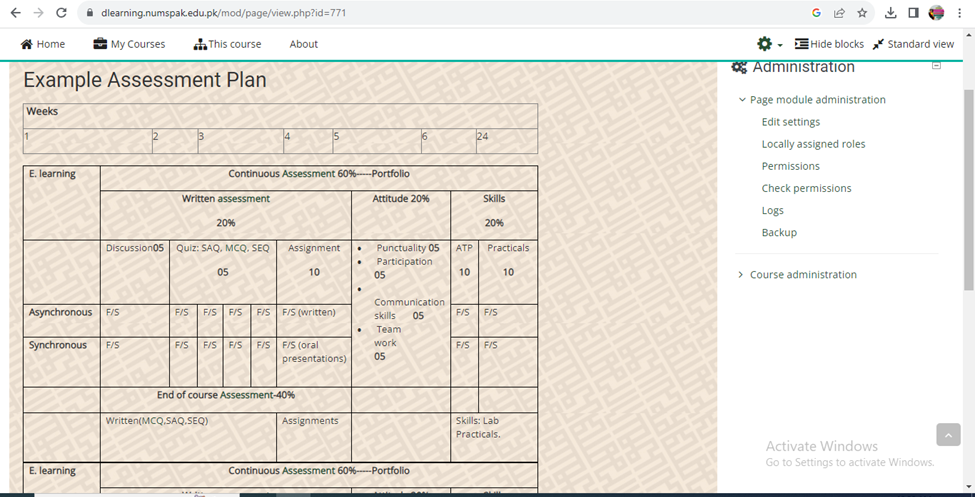 | | | |
| 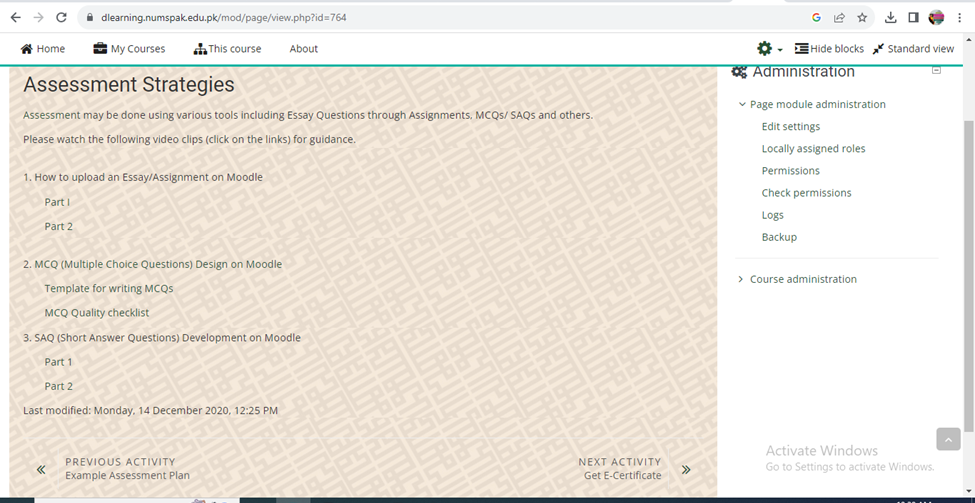 | | | |
| 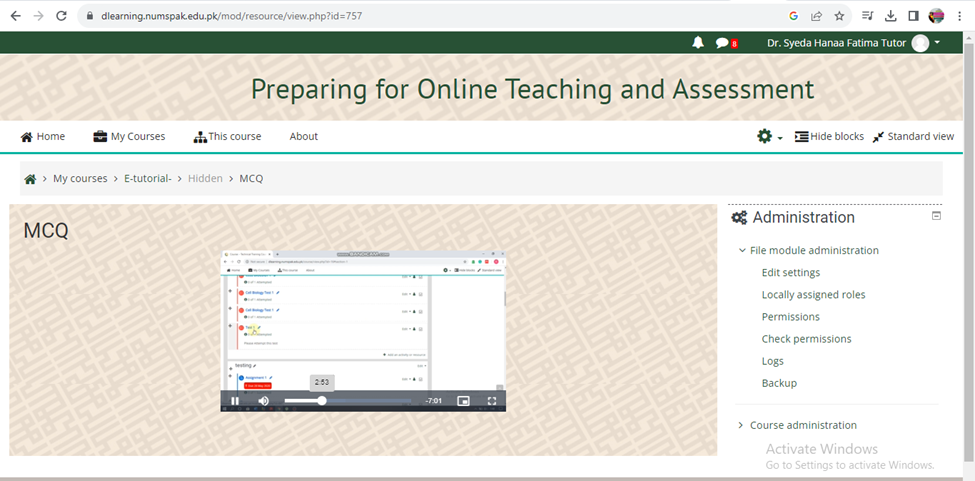 | | | |
| 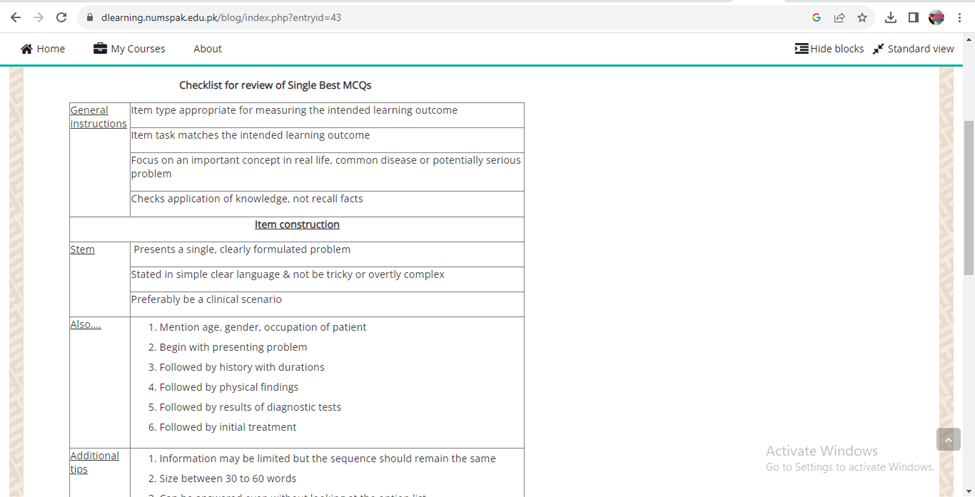 | | | |
| **New In:** | Knowledge of new software’s and apps that can help in effective learning | | |
| 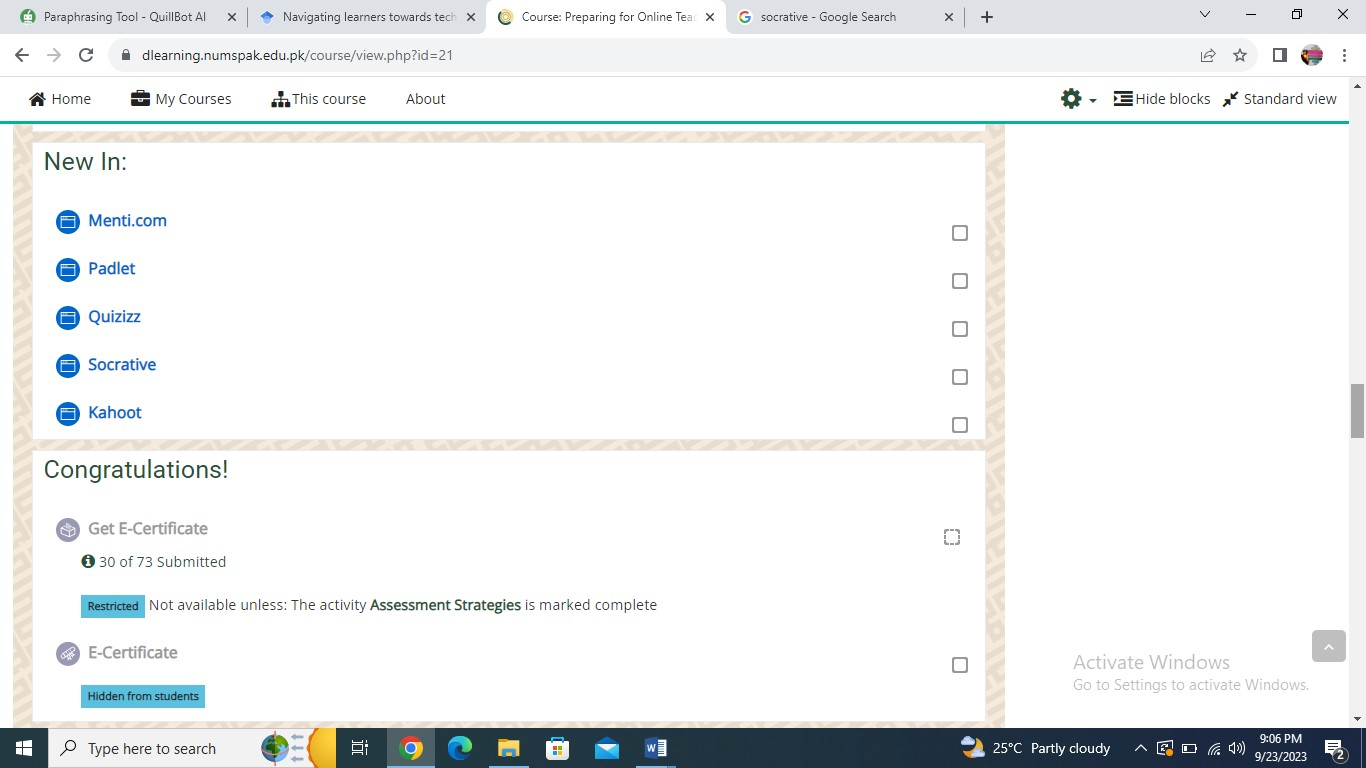 | | | |
| **E-Certificate** | Participants once completed the training were able to print their Certificates | | |
| 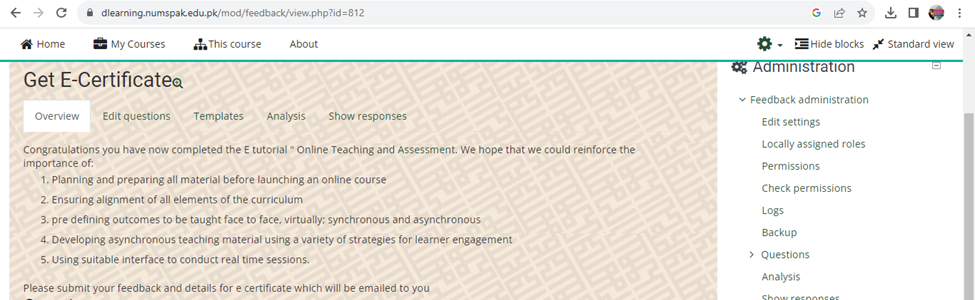 | | | |
| Throughout these stages, participants engaged in a structured and thoughtful learning process, enabling them to enhance their teaching skills and contribute effectively to the field of education. | | | |

**Survey form**

**“Empowering Health Professions Educators: Enhancing Curriculum Delivery through Customized E-Tutorial Training on Fundamental Digital Tools”**

Dear Participants, we are conducting this survey to assess the preparedness of faculty in designing and implementing e-learning through a preliminary KAP survey. This will be followed by evaluation of the impact of an educational intervention based on results of this survey.

Filling this form will be considered your consent. It will be used only for academic/research purposes and anonymity of the data will be maintained.

1. **Participant Details**

Name (optional)____________________________________

Age

- 24-35
- 36-45
- 46-55
- 55-65

Gender

- Male
- Female

Designation

- Lecturer
- Assistant professor
- Associate Professor
- Professor

Specialty/subject _______________________

Years of teaching

- 5-10 years
- 11-15
- 16-20
- 21-25
- 26-30
- More than 30

Institution ____________________________

___________________________________________________________________________

1. **Choose the best possible description for the following:**
2. **Distance learning is**

- Use of technology to update student’s knowledge through online learning
- Use of technology to address distance between student and teacher
- Another name for E-learning

1. **E learning is**

- Simply a broadcast of documents in electronic format to students via the Internet
- Another name for distance learning
- An online communication between the teacher and the student which can be used in a classroom or an online setting.

1. **In Synchronous teaching**

- There is time gap between the instructions provided and response of the learners.
- There is defined time limit given by teacher to submit responses.
- Instructions are provided on the spot, as in face-to-face teaching.

1. **In an asynchronous teaching**

- There is time gap between the instructions provided and response of the learners.
- Instructions are provided on the spot, as in face-to-face teaching.
- Both teacher and student must be online at the same time

1. **Self-Study is**

- Study of topics as directed by teacher and is time bound
- Non-timetabled study hours spent outside the classroom for learning purposes e.g. for assignments, group work, class test
- A scheduled time to study within classroom for learning purposes

1. **Please rate yourself on the following items on a scale of 1-5, where**

**1=Poor** Have No knowledge & No practical experience of the subject

**2=Fair** Have knowledge but No practical experience of the subject

**3=Average** Have knowledge and practical experience but require some guidance

**4= Good** Have knowledge and practical experience, do not require guidance

**5= Excellent** Have knowledge & practical experience with the ability to start new initiative

1. Using technology and available soft wares for designing an online course

1=Poor 2=Fair 3= Average 4= good 5= Excellent

1. Have Knowledge of all elements of the curriculum that should be included in an online course

1=Poor 2=Fair 3= Average 4= good 5= Excellent

1. Designing a synchronous teaching session (Real time)

1=Poor 2=Fair 3= Average 4= good 5= Excellent

1. Designing an asynchronous teaching session (preparing a session)

1=Poor 2=Fair 3= Average 4= good 5= Excellent

1. Designing student assessment online

1=Poor 2=Fair 3= Average 4= good 5= Excellent

1. Conducting a synchronous teaching session (Real time)

1=Poor 2=Fair 3= Average 4= good 5= Excellent

1. Conducting an asynchronous teaching session (prepared and uploaded)

1=Poor 2=Fair 3= Average 4= good 5= Excellent

1. Conducting student assessment online

1=Poor 2=Fair 3= Average 4= good 5= Excellent

1. Providing feedback to students online

1=Poor 2=Fair 3= Average 4= good 5= Excellent

1. Uploading learning resources on web portal

1=Poor 2=Fair 3= Average 4= good 5= Excellent

**Using:**

1. Google classroom

1=Poor 2=Fair 3= Average 4= good 5= Excellent

1. Zoom

1=Poor 2=Fair 3= Average 4= good 5= Excellent

1. Microsoft teams

1=Poor 2=Fair 3= Average 4= good 5= Excellent

1. WhatsApp

1=Poor 2=Fair 3= Average 4= good 5= Excellent

1. Moodle

1=Poor 2=Fair 3= Average 4= good 5= Excellent

1. **Please give your agreement on the following items on a scale of 1-5, where**
2. **Strongly Disagree**
3. **Disagree**
4. **Neutral**
5. **Agree**
6. **Strongly agree**
7. **E Learning should be made part of regular student teaching.**

1=Strongly Disagree 2 =Disagree 3=Neutral 4=Agree 5= Strongly Agree

1. **E learning should be used for student teaching in addition to face to face sessions**

1=Strongly Disagree 2 =Disagree 3=Neutral 4=Agree 5= Strongly Agree

1. **I will use e learning to compliment my teaching even after COVID threat is over**

1=Strongly Disagree 2 =Disagree 3=Neutral 4=Agree 5= Strongly Agree

1. **Issues**
2. **Select the administrative issues that you faced:**

- Absence of e- learning policy
- Absence of designated faculty time
- Faculty resistance
- Absence of accountability and record keeping
- Mal distribution of faculty workload
- Lack of Interdepartmental coordination
- Scheduling issues of e learning sessions
- None
- Any other _____________________________________________

1. **Select the technical issues that you faced**

- Non-Availability of training program prior to the launch of e-learning
- Poor connectivity
- Non availability of Campus management system (LMS/CMS)
- Absence of technical support
- Non availability of Hardware
- None
- Any other _______________________________________________

1. **Select the academic issues that you faced**

- Lack of Courses /content availability
- Lack of Students active participation during teaching sessions
- Lack of faculty enthusiasm during teaching sessions
- Lack of Effective Assessment strategies
- Poor adaptability of teachers to e-learning
- Poor adaptability of students to e- learning
- None
- Any other _____________________________________

Summary of the survey form and section wise objectives

Section A – Participants’ Demographic details

Section B – Participants’ Knowledge

Section C – Participants’ Practices

Section D – Participants’ Attitude

Section E - Issues faced
